# HIV-1 Subtypes and 5′LTR-Leader Sequence Variants Correlate with Seroconversion Status in Pumwani Sex Worker Cohort

**DOI:** 10.3390/v10010004

**Published:** 2017-12-23

**Authors:** Raghavan Sampathkumar, Joel Scott-Herridge, Binhua Liang, Joshua Kimani, Francis A. Plummer, Ma Luo

**Affiliations:** 1 Department of Medical Microbiology, University of Manitoba, Winnipeg, MB, R3E 0J9, Canada; raghavans1@gmail.com (R.S.); jkimani@csrtkenya.org (J.K.); Francis.Plummer@umanitoba.ca (F.A.P.); 2 National Microbiology Laboratory, Public Health Agency of Canada, Winnipeg, MB, R3E 3R2, Canada; jascottherridge@gmail.com (J.S.-H.); Binhua.Liang@umanitoba.ca (B.L.); 3 Department of Biochemistry and Medical Genetics, University of Manitoba, Winnipeg, MB, R3E 0J9, Canada; 4 Department of Medical Microbiology, University of Nairobi, Nairobi, Kenya

**Keywords:** HIV/AIDS, seroconversion, 5′LTR-leader sequence, genetic diversity, primer binding site sequences, splicing donor sequences, packaging signal, HIV subtypes

## Abstract

Within the Pumwani sex worker cohort, a subgroup remains seronegative, despite frequent exposure to HIV-1; some of them seroconverted several years later. This study attempts to identify viral variations in 5′LTR-leader sequences (5′LTR-LS) that might contribute to the late seroconversion. The 5′LTR-LS contains sites essential for replication and genome packaging, viz, primer binding site (PBS), major splice donor (SD), and major packaging signal (PS). The 5′LTR-LS of 20 late seroconverters (LSC) and 122 early seroconverters (EC) were amplified, cloned, and sequenced. HelixTree 6.4.3 was employed to classify HIV subtypes and sequence variants based on seroconversion status. We find that HIV-1 subtypes A1.UG and D.UG were overrepresented in the viruses infecting the LSC (*P* < 0.0001). Specific variants of PBS (*Pc* < 0.0001), SD1 (*Pc* < 0.0001), and PS (*Pc* < 0.0001) were present only in the viral population from EC or LSC. Combinations of PBS [PBS-2 (*Pc* < 0.0001) and PBS-3 (*Pc* < 0.0001)] variants with specific SD sequences were only seen in LSC or EC. Combinations of A1.KE or D with specific PBS and SD variants were only present in LSC or EC (*Pc* < 0.0001). Furthermore, PBS variants only present in LSC co-clustered with PBS references utilizing tRNA^Arg^; whereas, the PBS variants identified only in EC co-clustered with PBS references using tRNA^Lys,3^ and its variants. This is the first report that specific PBS, SD1, and PS sequence variants within 5′LTR-LS are associated with HIV-1 seroconversion, and it could aid designing effective anti-HIV strategies.

## 1. Introduction

In 2016, there were 62,000 new HIV infections, and 1,600,000 people living with HIV in Kenya [1]. Efforts are underway, globally, to find ways to prevent infection, as well as to explore practical cures for HIV [2,3]. The most at-risk individuals for infection by HIV are commercial sex workers (CSW), intravenous drug users, and men who have sex with men (MSM). The CSW population is at increased risk, as they may have hundreds of sexual partners each year. Compounding the risk of infection and transmission, many of them could be intravenous drug users, and/or may be infected with other sexually transmitted pathogens that could enhance HIV transmission [4]. Kenya has around 133,675 sex workers [1]. The percentage of female sex workers of a population has been reported to strongly correlate with total HIV/AIDS prevalence [4]. Female sex workers have a 13.5-fold higher risk of being HIV infected compared to other women [5]. The high infection risk of this population can provide critical insights into HIV infection, disease progression, and transmission. The research on the HIV infection of this group could provide clues for designing effective HIV-1 control strategies.

The Pumwani sex worker cohort in Kenya was established in 1985. The overall HIV-1 prevalence in the cohort is over 73.7%. The majority of HIV-1 negative women at cohort entry seroconverted within three years. A small group of women remain HIV-1 negative, despite heavy exposure through active sex work [6,7]. This observed resistance to HIV-1 infection was not due to safer sexual practices, altered cellular susceptibility to HIV-1, or known chemokine-receptor polymorphisms [8]. Polymorphism in HLA and non-HLA genes have been shown to influence HIV-1 resistance in this cohort [7,9,10,11,12,13,14,15], as well as in other populations [16]. Some of the HIV “resistant” sex workers seroconverted after being seronegative for many years; these women were designated as late seroconverters (LSC). Late seroconversion may occur in HIV-1-resistant sex workers, despite preceding HIV-specific CD8^+^ T cell responses [17]. Viral factors, such as subtype and functional genetic differences, have not been properly examined. It is conceivable that these women, previously resistant to HIV-1 infection, were infected by a more infectious, pathogenic viral species. This study intends to examine the viral factors infecting this group of late seroconverters.

Among the major groups of HIV-1, M, N, O, and P, M group viruses have been responsible for the majority of HIV-1 infections worldwide [18]. Nine major subtypes identified within group M viruses are A-D, F-H, J, and K. Sub-subtypes have been observed for clade A (A1, A2) as well as F (F1, F2) viruses. Additionally, group M includes 90 circulating recombinant forms. Globally, more prevalent subtypes are B (56.0%), C (17.0%), A (5.8%), D (3.1%), G (1.0%) and F (1.0%). In Kenya, subtypes A (68.0%), D (13.5%), and C (5.8%) were more common [19]. Analysis of 41 near full-length HIV-1 sequences from Kenya reported 56.1% subtype A, 2.4% each of subtypes C and D, and 39% recombinant [20]. Another study of 176 Kenyan patients observed 73.9% A1, 10.8% C, 10.2% D, and 0.6% of G and A2 clades [21]. Our previous analysis of HIV gag of 468 HIV-1 positive women also showed that the Pumwani sex worker cohort is primarily infected with clade A1 at 71%, 65%, 67%, and 63%, followed by clade D at 14%, 22%, 23%, and 20% for p17, p24, p7, and p6, respectively [22]. Different subtypes exhibit unique influences over viral transmission, replication, disease progression, virulence, and susceptibility to antiretroviral drugs [23,24,25,26]. Studies have shown that debilitated HIV-1 viruses needed only few mutations to attain fitness recovery, and these events most commonly involved the 5′ untranslated leader sequence [27]. This region contains three important sites for viral replication: primer-binding site (PBS), major splice donor site (SD), and major packaging signal (PS) [28,29]. HIV-1 loses infectivity upon complete deletion of PBS, and this highlights the functional importance [30]. Reverse transcription initiation involves the binding of cellular tRNA^Lys,3^ to the 18 nucleotide PBS that is located upstream of *gag*. This sequence is complementary to the 3′ terminal 18 nucleotides of this particular tRNA molecule [31]. Usage of tRNA^Lys,5^, though infrequent, as primer in HIV replication, has also been reported [32]. While all retroviruses make use of tRNA as a primer for reverse transcription, specific viruses are known to exhibit preference for particular tRNA molecules, as exemplified by usage of tRNA^Pro^ and tRNA^Trp^ by murine leukemia virus and avian sarcoma virus-avian leukosis virus groups, respectively [33]. The preference for usage of specific tRNA primers by HIV-1 for reverse transcription could be linked to its fitness [34]. HIV-1 splicing aids in optimal expression of its proteins, facilitating infection and subsequent generation of new infectious viral progenies. HIV-1 uses several splice sites in its genome to produce more than 40 different mRNA transcripts [35]. Major splice donor site, SD1, joins to a 3′ splice acceptor region downstream of *pol*, and this results in a transcript encoding envelope glycoproteins. Furthermore, all downstream splicing events become silenced if SD1 is mutated [36]. The PS is a GGAG tetraloop found at the end of stem–loop SL3 [37]. This structure is seen only among unspliced HIV-1 RNAs, and it interacts with nucleocapsid protein, a breakdown product of gag polyprotein [37]. It has been suggested that the newly synthesized gag protein could bind PS, leading to translation inhibition of part of unspliced RNAs, which in turn might ensure full-length viral RNA molecules are available for packaging [38]. Deletions in PS are known to cause substantial reduction in genome packaging capability [29]. Primer-binding site, SD1, and PS, are therefore pivotal sequence elements for the replication and proliferation of HIV-1.

The presence of these three essential sites in 5′LTR-leader sequence led us to choose this region to examine the viruses infecting the late seroconverters. We compared 5′LTR-leader sequences from the late seroconverters with those from women who were seropositive at enrollment, or seroconverted within the first three years of enrollment in the Pumwani sex worker cohort. We hypothesized that late seroconverters were infected with specific variants of HIV-1, whose distinct 5′ leader sequence profile could confer potential replicative advantages, besides efficient genome packaging capability.

## 2. Materials and Methods

### 2.1. Sample Collection

HIV-1 positive sex workers and late seroconverters from the Pumwani sex worker cohort were selected for this study. No anti-retroviral treatments (ARTs) were available during the sample collection period in Kenya, thus, none of the samples analyzed in this study were confounded by ARTs. Informed written consent was obtained from all study subjects. The University of Manitoba, as well as University of Nairobi ethics review panels, have approved studies with these subjects. Women in this cohort are routinely screened for HIV-1 infection by serology and PCR amplification for the *env*, *nef*, and *vif* genes. Women were defined as resistant to HIV-1 infection if they remain HIV-1 seronegative and PCR negative for a minimum of three years of follow up after enrollment [6]. The late seroconverters were defined as those who seroconverted after meeting the defined resistance criteria [6,17]. In this study, 20 patients met this criterion. Seven of these patients also had samples collected at different dates since seroconversion. The control population consisted of 122 seropositive patients, of which 101 women were positive at enrollment, and 21 seroconverted within three years after enrollment. Sixteen control subjects had more than one timepoint sample. The average seronegative time of the late seroconverters is 5.94 ± 2.92 years, compared to an average of 0.80 ± 0.70 seronegative years of the 21 seroconverters in the positive control group.

### 2.2. Genomic DNA Isolation and Nested PCR Amplification of Partial 5′LTR of HIV-1

**Figure 1:**
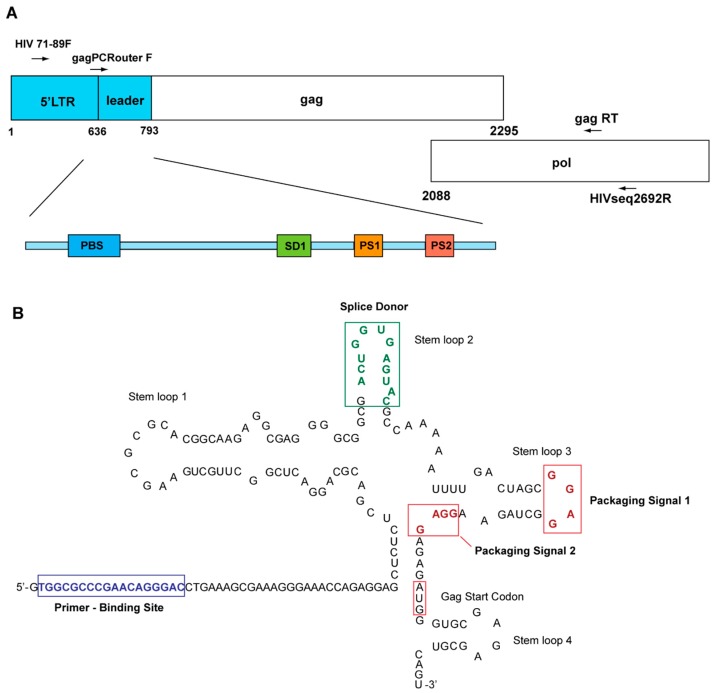
HIV-1 5′LTR leader sequences. (**A**). A schematic sketch of 5′LTR-leader sequence variant positions analyzed in this study; (**B**). a schematic sketch of secondary structure of HIV-1 5′leader sequence.

Genomic DNA was isolated from peripheral blood mononuclear cells of the study subjects using QIAamp DNA Mini Kit (Qiagen Inc., Mississauga, ON, Canada). Nested PCR was carried out, using Expand High Fidelity PCR system (Roche Diagnostics, Mannheim, Germany), to amplify a 2 kb fragment containing partial 5′LTR, HIV-1 *gag*, and partial protease gene (found in *pol*) (Figure 1A,B). Primers HIV71-89F (5′-CTTCCCTGATTGGCAGAAY-3′) and HIVseq2692R (5′-GGATTTTCAGG CCCAATTTTTG-3′) were used for the first round of amplification. The PCR cycle conditions were 2 min initial denaturation at 94 °C, followed by 35 cycles of 15 s at 94 °C, 30 s at 53 °C, and 68 °C for 5 min, with final extension at 68 °C for 15 min. Primers Gag PCR outerF (5′-AATCTCTAGCAGTGGCGCCCGAACAG-3′) and GagRT (5′-CCATTGTTTAACCTTTGGGCCATCCA-3′) were used for the second round PCR reaction. One microliter of PCR product from first round amplification was used as template. Thermal cycler parameters were set as 94 °C for 2 min, 35 cycles of 94 °C for 15 s, 59 °C for 30 s and 68 °C for 4 min, with final extension at 68 °C for 10 min. All PCR products were examined using 1% agarose gel electrophoresis.

### 2.3. Cloning and Sequencing of Amplified Partial 5′LTR Sequences

Prior to cloning, the PCR products were TA extended. Each TA extended PCR product was ligated into pCR®4-TOPO® vector (TOPO TA Cloning Kit for Sequencing, Invitrogen Life Technologies, Carlsbad, CA, USA) and transformed into One Shot® TOP10 Chemically Competent *E. coli*. Forty-eight clones were picked from each sample and cultured for 16–20 h in 2 mL LB medium with ampicillin (200 μg/mL). Bacteria cultures were pelleted by centrifugation for 6 min at 1900 *g*. QIAprep 96 Turbo Miniprep Kit protocol was used to isolate plasmids containing the amplified HIV-1 fragment. EcoR1 restriction digestion and agarose gel electrophoresis were conducted to detect the presence of insert DNA. T3 and T7 sequencing primers were used to sequence the clones using BigDye version 3.1 Cycle sequencing kit (Applied Biosystems^TM^, Carlsbad, CA, USA), and analyzed with an ABI3730XL DNA Analyzer, available at DNACORE facility of the National Microbiology Laboratory, Winnipeg, MB, Canada.

### 2.4. Sequence and Phylogenetic Analyses

The sequences were examined using Sequencher version 4.6 (Gene Codes Corporation, MI, USA). HIV *gag* sequences were removed and 160 nucleotide sequences of partial 5′LTR region, including the part of *U5* and untranslated leader sequence, were retained for further analysis. Close to 4000 5′LTR leader sequences have been generated. Phylogenetic analysis using MEGA 3.1 [39] was done to classify viral subtypes. Briefly, partial 5′LTR sequences were aligned with 51 reference sequences obtained from HIV sequence database [19]. Alignment was done with ClustalW and phylogenetic trees were generated. Alignment and phylogenetic relatedness to reference sequences permitted subtype identification for each clone. To confirm the subtype assignment of 5′LTR sequences by phylogenetic analysis, we also conducted phylogenetic analysis of p17 sequences of these cloned sequences. The results confirmed the subtype assignment using the sequences of the partial 5′LTR region. Two examples of the phylogenetics analysis using p17 sequences are shown in Supplemental Figures S1 and S2. To assess the possible function of PBS variants observed in the present study, 19 published PBS sequences, that used different tRNA primers for reverse transcription, were taken as reference to construct a maximum likelihood method based phylogenetic tree, using MEGA 6. The 19 PBS sequences in the reference alignment included those corresponding to tRNA^Lys,3^ (wild-type), tRNALys^1,2^, tRNA^Lys,5a^, tRNA^Lys,1^, EctRNA^Lys,3^ (*E. coli* tRNA), tRNA^Pro^, tRNA^Ile^, tRNA^Met^, tRNA^Met(e)^ (used in elongation), tRNA^Met(i)^ (used in initiation), tRNA^Met(i)^AG (contains a transition), tRNA^Ser^, tRNA^Phe^, tRNA^Thr^, tRNA^Gln,1^, tRNA^Gln,3^, tRNA^His^, tRNA^Arg(ACG)^ and tRNA^Arg(CCU)^ [32,34,40,41,42,43,44,45,46,47].

### 2.5. Sequence Variant Classification by Recursive Partitioning Analysis

Recursive partitioning methods have become popular and widely used tools for non-parametric regression and classification in many scientific fields [48]. They can deal with large numbers of predictor variables, even in the presence of complex interactions, and have been applied successfully in genetics, clinical medicine, and bioinformatics within the past few years [48]. In this study, we used the recursive partitioning methods based interactive tree analysis tool in HelixTree SNP and Variation Suite version 6.4.3 (Golden Helix, Inc., Bozeman, MT, USA) to analyze the large pool of sequence variants of the three important sites (PBS, SD, and PS) within the 5′LTR leader region. The interactive tree analysis tool was developed based on formal inference recursive modeling (FIRM) technology by Dr. Douglas Hawkins [48,49,50,51,52,53,54,55,56,57] accessed 21 December 2017), and has taken the statistical foundations of FIRM and augmented it with faster and more exact segmenting algorithms. It has also extended FIRM methods to include multivariate response. Recursive partitioning uses a set of data and, based on some criterion, partitions or splits the original set into smaller sets. These smaller sets are, in turn, split into still smaller sets. This process continues (recursively) until additional splitting of the data into smaller sets gives no statistically meaningful information.

For example, because the aim of our study is to identify the sequence variants of the three sites within the HIV 5′LTR leader region that are predominantly detected among late seroconverters, we designated the *u*-value of late seroconverters as 1.0 and the *u*-value for early seroconverters as 0. For example, when analyzing sequence variants of PBS using the tree analysis tool, the sequence variants were partitioned based on whether they are detected in the early or late seroconverters and the *p* value. PBS sequence variants in the tree node with *u*-value equal to 1 indicate that the PBS sequence variants were identified only in late seroconverters, whereas the PBS sequence variants in the tree node with *u*-value equal to 0 were only identified in early seroconverters. The PBS sequence variants in the tree nodes with *u*-value varying between 1 and 0 indicate that the sequences exist in both early and late seroconverters. Because it is possible that not only specific sequence variants of PBS can influence seroconversion, but also the combinations of the PBS sequence variants with specific sequences of SD or PS may play a role in seroconversion, the PBS sequences in the nodes with *u*-values between 0 and 1 can be further classified by sequence variants of SD or PS. At each step of analysis, a combination of *u*-value and *p* value was used to define the sequences associated with late or early seroconverters.

Differences in subtype distributions of the sequence variants between late seroconverters and controls were analyzed by Pearson χ^2^ analysis using SPSS version 13.0. *p* values equal to or less than 0.05 were considered statistically significant.

## 3. Results

### 3.1. Uganda A1 and D Subtype 5′LTR-Leader Sequences Were Significantly Enriched in HIV Viral Population from Late Seroconverters

**Figure 2:**
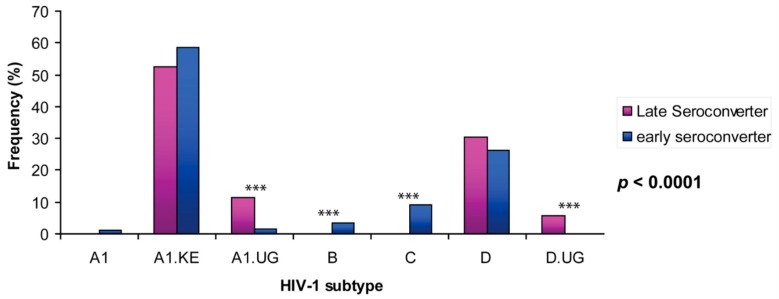
HIV-1 subtype distribution among late seroconverters and early seroconverters. *** : *p* value is less the 0.0001.

A total of 3678 sequences from 20 late seroconverters and 122 early seroconverters were phylogenetically analyzed to determine their HIV-1 subtypes. This analysis only included the sequences of the earliest sampling date of the available samples from each patient. Similar to previous studies, subtype A predominates in the HIV viral population of this Kenyan population, followed by subtype D. The frequencies of subtypes A1.KE, A1.UG, D, and D.UG were 57.2%, 3.7%, 27.2%, and 1.3%, respectively (Figure 2 and Table 1). There is a significant difference in overall subtype distribution of 5′LTR leader sequences between viral population in early and late seroconverters (*p* < 0.0001). While subtypes B (0% versus 3.4%, *p* < 0.0001) and C (0% versus 9.2%, *p* < 0.0001) sequences were not observed among the late seroconverters, subtype A1.UG sequences were significantly enriched in the late seroconverters compared to the ones in early seroconverters (11.4% versus 1.5%, *p* < 0.0001). Further, subtype D.UG sequences were absent in early seroconverters (5.7% versus 0%, *p* < 0.0001). It is apparent that the viral population infecting late seroconverters was enriched with subtype A1.UG and D.UG 5′LTR leader sequences.

### 3.2. Unique Sequences and Combinations of PBS, SD, and Ps Sequences in Late Seroconverters

**Figure 3:**
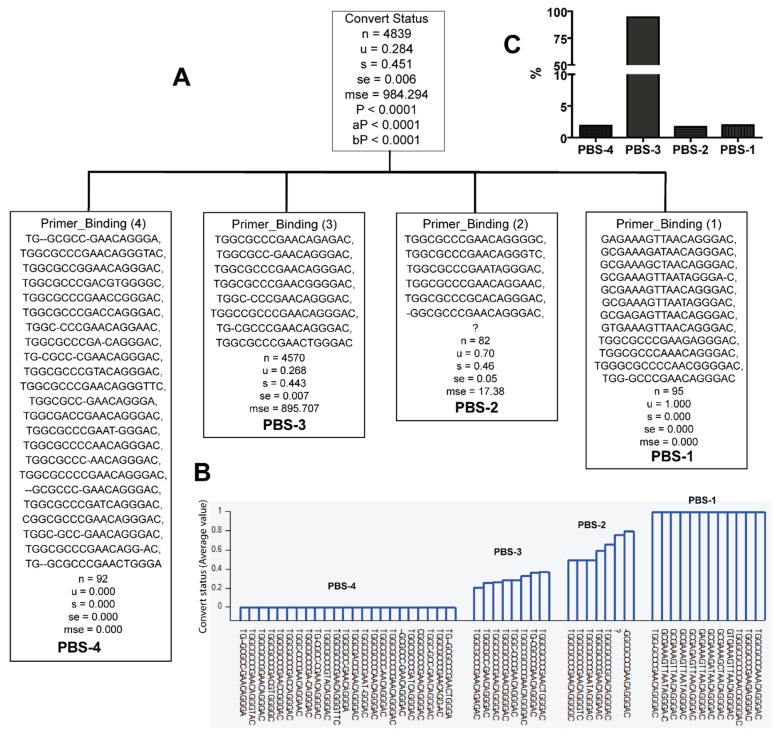
Classification of primer binding sequence (PBS) variants according to whether they were identified from late seroconverters (*u* = 1) or early seroconverters (*u* = 0). Note: question mark denotes lack of sequence. *u—*mean value; s—standard deviation; se—standard error; mse—mean square error; *p—p* value; aP—adjusted *p* value; bP—Bonferroni corrected *p* value. (**A**) Classification tree; (**B**) PS variants and their distribution with seroconversion status; (**C**) PBS variants frequencies.

**Figure 4:**
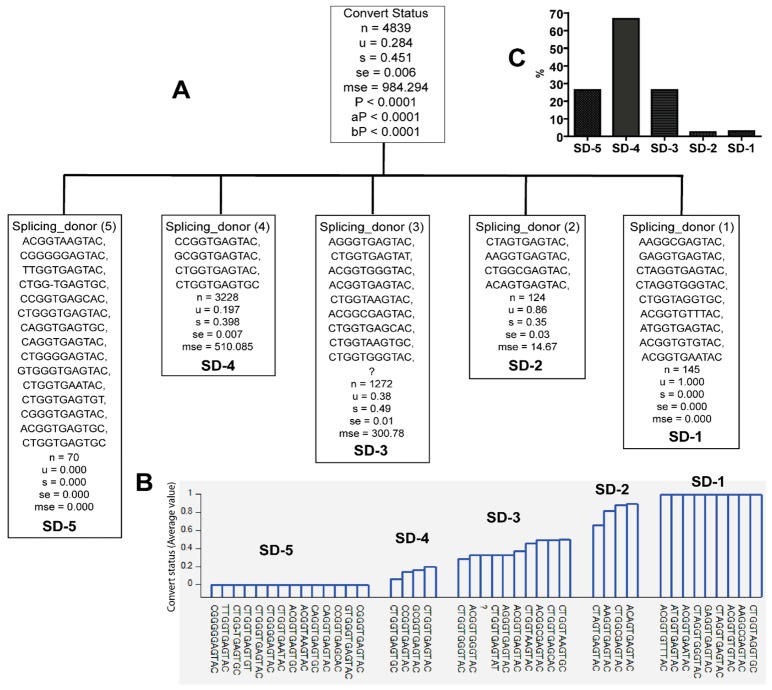
Classification of splicing donor (SD) sequence variants according to whether they were identified from late seroconverters (*u* = 1) or early seroconverters (*u* = 0). Note: question mark denotes lack of sequence. *u—*mean value; s—standard deviation; se—standard error; mse—mean square error; *p*—*p* value; aP—adjusted *p* value; bP—Bonferroni corrected *p* value. (**A**) Classification tree; (**B**) PS variants, and their distribution with seroconversion status; (**C**) SD variants frequencies.

**Figure 5:**
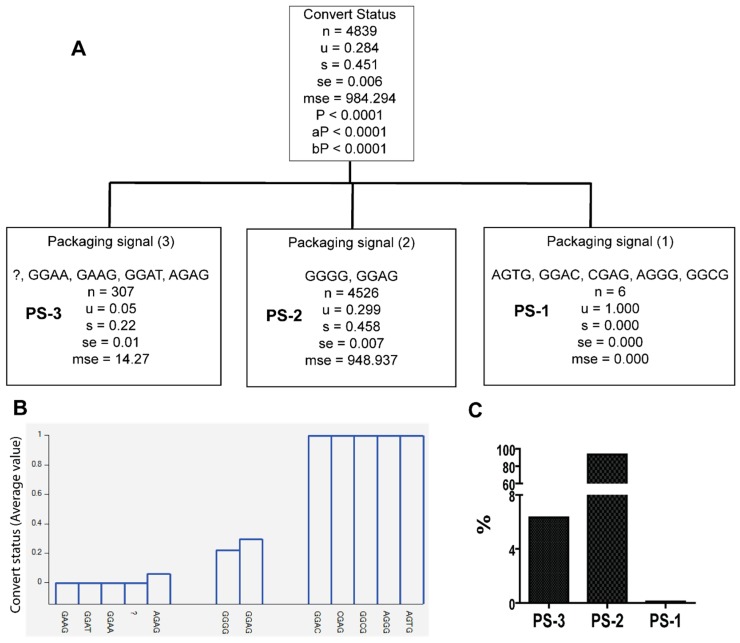
Classification of packaging signal (PS) sequence variants according to whether they were identified from late seroconverters (*u* = 1) or early seroconverters (*u* = 0). Note: question mark denotes lack of sequence. *u—*mean value; s—standard deviation; se—standard error; mse—mean square error; *p—p* value; aP—adjusted *p* value; bP—Bonferroni corrected *p* value. (**A**) Classification tree; (**B**) PS variants and their distribution with seroconversion status; (**C**) PS variants frequencies. “?” indicate the absence of the sequences”.

We then examined whether specific sequences of primer binding site (PBS), splice donor (SD), and packaging signal (PS), and their combinations, are more likely to be associated with HIV viral population in late seroconverters. For this, we included all 4839 sequences from multiple sample dates of the patients in recursive partition analysis using the Tree analysis tool of HelixTree 6.4.3. Recursive partitioning analysis classifies the 5′LTR-leader sequence variants based on their nucleotide sequences, subtypes, and their origin, into early (designated as 0) or late seroconverters (designated as 1) (Figure 3, Figure 4 and Figure 5 and Table 2). The analysis showed that specific sequence variants of PBS were only identified in the viral population of either early or late seroconverters (*Pc* < 0.0001) (Figure 3 and Table 2). Specifically, 12 PBS sequence variants were only found in the viral population of late seroconverters (PBS-1, Figure 3 and Table 2), and 23 PBS sequence variants were only identified in the viral population of early seroconverters (PBS-4, Figure 3A,B, and Table 2). Some PBS sequence variants were identified in the viral population of both early and later seroconverters (PBS-2 and PBS-3, Figure 3 and Table 2).

Similarly, specific sequence variants of SD were only identified in the viral population of either early or late seroconverters (*Pc* < 0.0001) (Figure 4 and Table 2). Nine SD sequence variants were only found in the viral population of late seroconverters (SD-1, Figure 4A,B and Table 2), while 14 SD sequence variants were only found in the viral population of early seroconverters (SD-5, Figure 4A,B and Table 2). Some SD sequence variants were identified in the viral population of early as well as late seroconverters (SD-2, 3, 4, Figure 4 and Table 2).

Likewise, specific sequence variants of PS were only identified in the viral population of either early or late seroconverters (*Pc* < 0.0001) (Figure 5 and Table 2). Five PS sequence variants were only identified in the viral population of late seroconverters (PS-1, Figure 5A,B and Table 2), while four PS sequence variants were only seen in the viral population of early converters (PS-3, Figure 5A,B and Table 2). Some PS sequence variants were identified in the viral population of both early and late seroconverters (PS-2, Figure 5A,B and Table 2).

**Figure 6:**
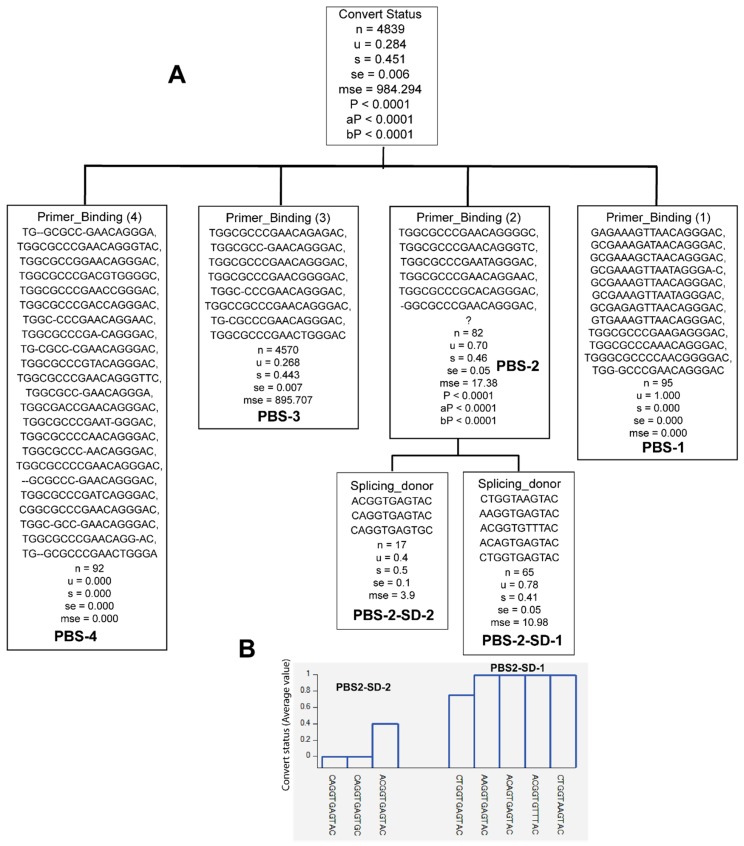
The combination of PBS variants (PBS-2) and splicing donor sequences identified only in either late (*u* = 1) or early seroconverters (*u* = 0). (**A**) PBS sequence variants in tree node of PBS-2 were further classified with SD sequence variants based on whether they were identified from late or early seroconverters; (**B**) the figure shows *u* value of sequence combinations based on whether the PBS-SD combinations were identified from late (1) or early (0) seroconverters. Note: *n—*counts (clone); *u—*mean value; s—standard deviation; se—standard error; mse—mean square error; *p—p* value; aP—adjusted *p* value; bP—Bonferroni corrected *p* value. “?” denotes lack of sequences”.

**Figure 7:**
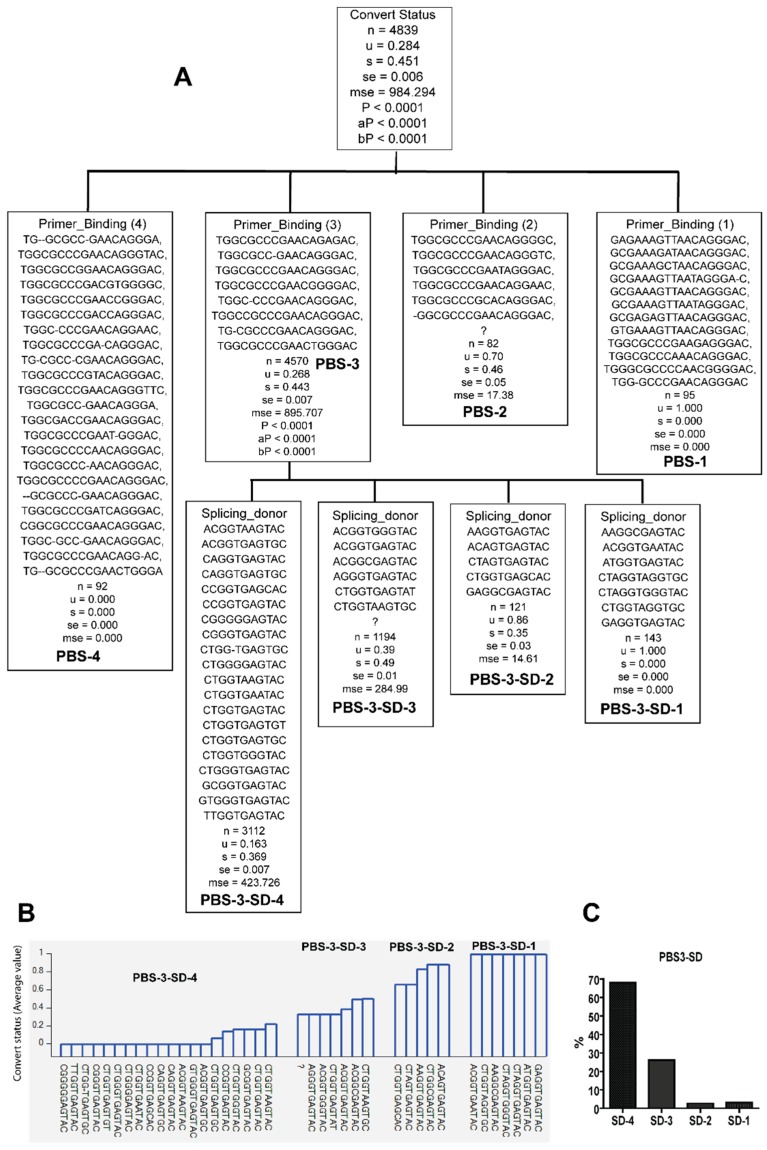
The combination of PBS variants (PBS-3) and splicing donor sequences identified only in either late (*u* = 1) or early seroconverters (*u =* 0). (**A**) PBS sequence variants in tree node of PBS-3 were further classified with SD sequence variants based on whether they were identified from late or early seroconverters. (**B**) The figure shows *u* value of sequence combinations based on whether the PBS–SD combinations were identified from late (1) or early (0) seroconverters. Note: *n—*counts (clone); *u—*mean value; s—standard deviation; se—standard error; mse—mean square error; *p—p* value; aP—adjusted *p* value; bP—Bonferroni corrected *p* value. (**C**) PBS3–SD variants frequencies. “?” denotes: lack of sequences”.

For the primer binding site sequence variants that existed in both early and late seroconverters (PBS-2 and PBS-3, Figure 3 and Table 2), we conducted further analysis to see whether combinations of specific PBS, SD sequence variants were more likely to exist in the viral population of late seroconverters. Further recursive analysis for the six sequence variants in the PBS-2 node with sequence variants of SD showed that the combinations of four specific SD sequence variants with the six PBS variants were only identified in the late seroconverters (PBS-2-SD-1; Figure 6A,B and Table 3). Similarly, PBS-3 node sequences in combinations with seven specific SD sequences occurred only in the viral population of late seroconverters (PBS-3-SD-1; Figure 7A,B and Table 3). In contrast, PBS-3 and 14 specific SD sequence variants (PBS-3-SD-4) existed only in the early seroconverters (Figure 7A,B and Table 3).

### 3.3. Combinations of Subtype A1.KE or D with Unique PBS and SD Sequence Variants in Late Seroconverters

**Figure 8:**
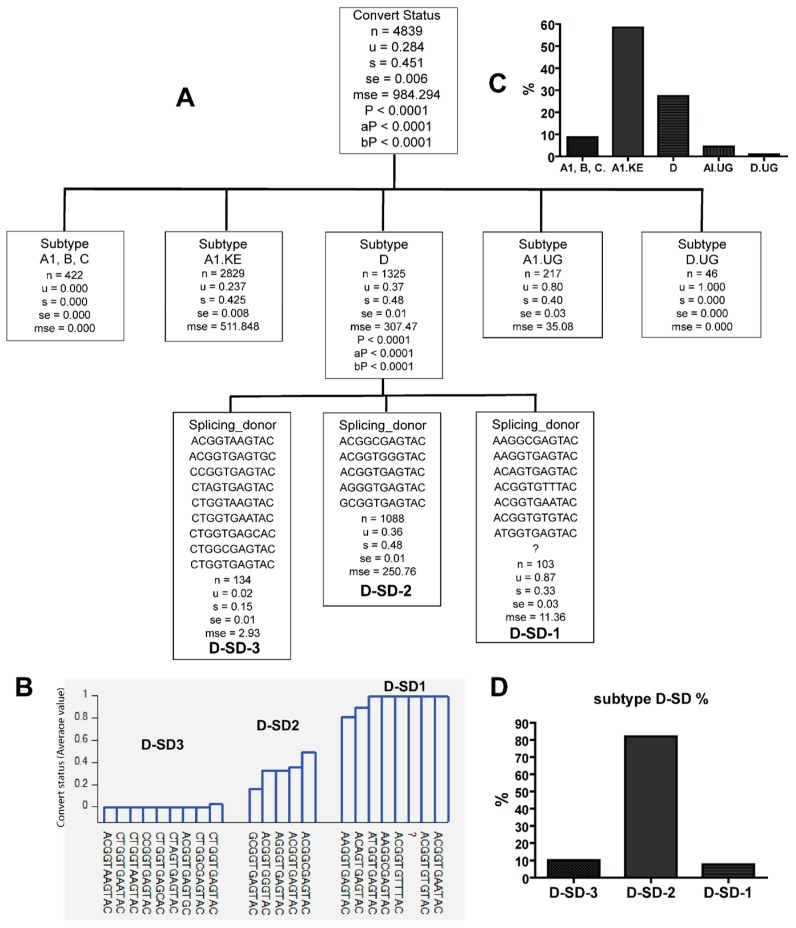
The combination of HIV subtype D and splicing donor sequences (SD) identified only in either late (*u =* 1) or early seroconverters (*u =* 0). (**A**) HIV subtype D in tree node of subtype D was further classified with SD sequence variants based on whether they were identified from late (*u =* 1) or early seroconverters (*u =* 0); (**B**) the figure shows *u* value of sequence combinations based on whether the subtype D–SD combinations were identified from late (1) or early (0) seroconverters. Note: *n—*counts (clone); *u—*mean value; s—standard deviation; se—standard error; mse—mean square error; *p—p* value; aP—adjusted *p* value; bP—Bonferroni corrected *p* value; (**C**) HIV subtype frequency; (**D**) Subtype D–SD frequencies in subtype D.

**Figure 9:**
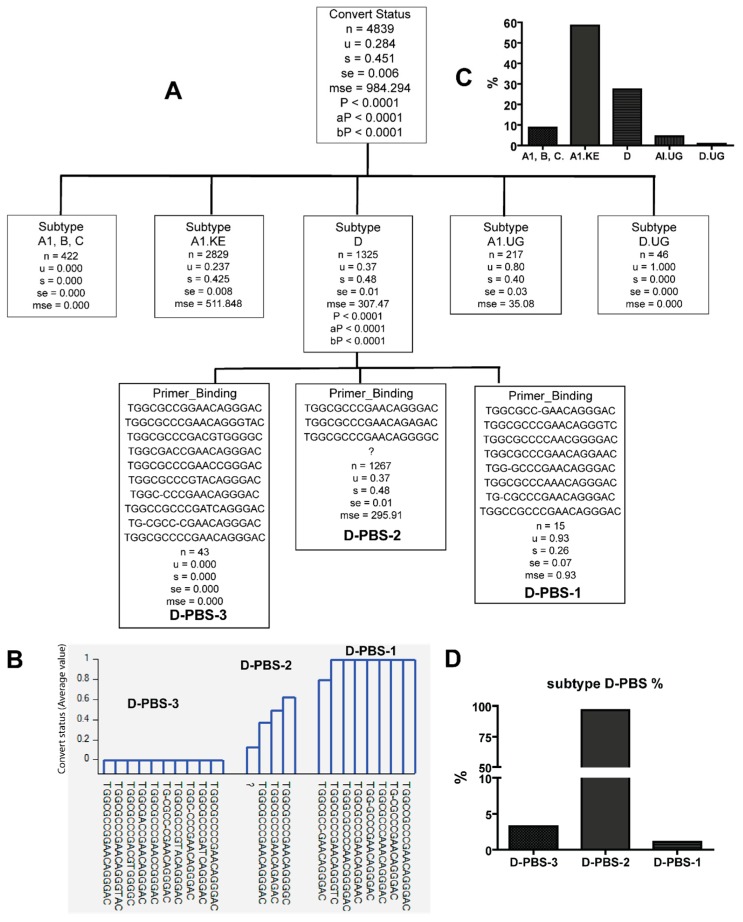
The combination of HIV subtype D and primer binding sequences (PBS) identified only in either late (*u =* 1) or early seroconverters (*u =* 0). (**A**) HIV subtype D in tree node of subtype D was further classified with PBS sequence variants, based on whether they were identified from late or early seroconverters. (**B**) The figure shows *u* value of sequence combinations based on whether the subtype D–PBS combinations were identified from late (1) or early (0) seroconverters. Note: *n—*counts (clone); *u*—mean value; s—standard deviation; se—standard error; mse—mean square error; *p—p* value; aP—adjusted *p* value; bP—Bonferroni corrected *p* value; “?” denotes the lack of sequence. (**C**) HIV subtype frequency. (**D**) Subtype D–PBS frequencies in subtype D.

**Figure 10:**
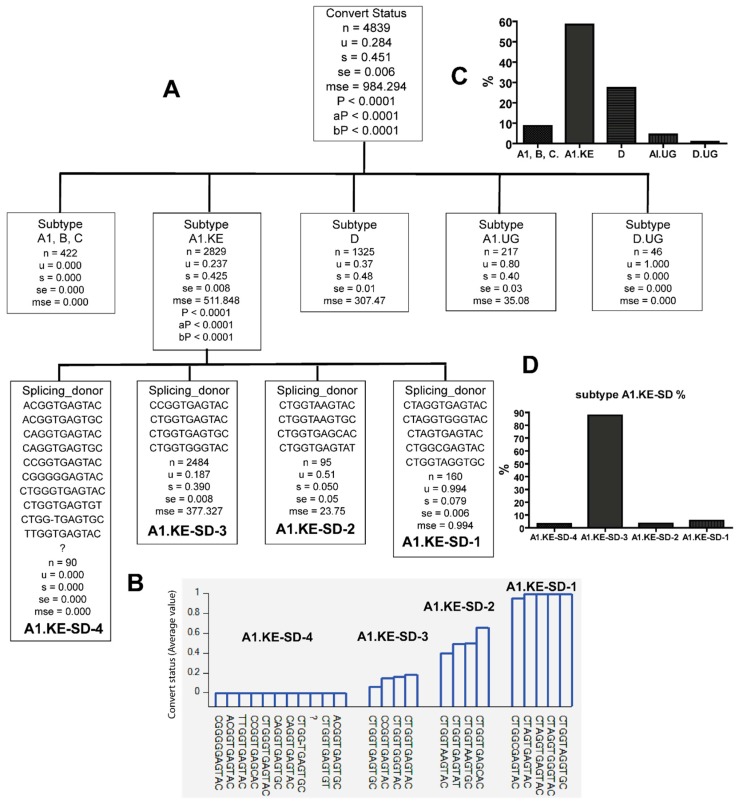
The combination of HIV subtype A1.KE and splicing donor sequences (SD) identified SD sequences only in either late (*u =* 1) or early seroconverters (*u =* 0). (**A**) HIV subtype A1.KE in tree node of subtype A1.KE was further classified with SD sequence variants based on whether they were identified from late or early seroconverters. (**B**) The figure shows *u* value of sequence combinations based on whether the subtype A1.KE-SD combinations were identified from late (1) or early (0) seroconverters. Note: *n—*counts (clone); *u—*mean value; s—standard deviation; se—standard error; mse—mean square error; *p—p* value; aP—adjusted *p* value; bP—Bonferroni corrected *p* value; “?”: denotes lack of sequence. (**C**) HIV subtype frequency. (**D**) Subtype A1.KE-SD frequencies in subtype A1.KE.

The late seroconverters are most likely to be infected with HIV variants with 5′LTR sequences belonging to A1.UG and D.UG, and specific PBS, SD, and PS variants are only identified in the viral population infecting the late seroconverters. However, late seroconverters were also infected with A1.KE and D, the two major HIV subtypes circulating in Kenya. Are there unique PBS, SD, and PS sequence variants in A1.KE and D infecting late seroconverters? The recursive analysis showed that specific SD variants or PBS variants in subtype D were identified only in late seroconverters or early seroconverters (Figure 8 and Figure 9, and Table 3). Specific SD variants in A1.KE were only identified in late seroconverters or early seroconverters (Figure 10, and Table 3). Thus, A1.KE or D with specific PBS and SD variants infect late seroconverters.

Our study showed that late seroconverters are more likely to be infected with A1 and D from Uganda, and specific PBS, SD, and PS sequences were only identified in the late seroconverters. Also, A1.KE and D with specific PBS and/or SD variants are also likely to infect late seroconverters. Table 4 summarized the identified 5′LTR subtypes, PBS, SD, PS variants, and the combinations identified and enriched in the 20 late seroconverters. These identified 5′LTR subtypes, PBS, SD, PS, and their combinations were identified and enriched in 16 out 20 late seroconverters (Table 4). The subtype classification of 5′LTR-leader sequence of viruses infecting late seroconverters is shown in Table 5.

### 3.4. Potential Functional Differences among PBS Variants in Late Seroconverters

**Figure 11:**
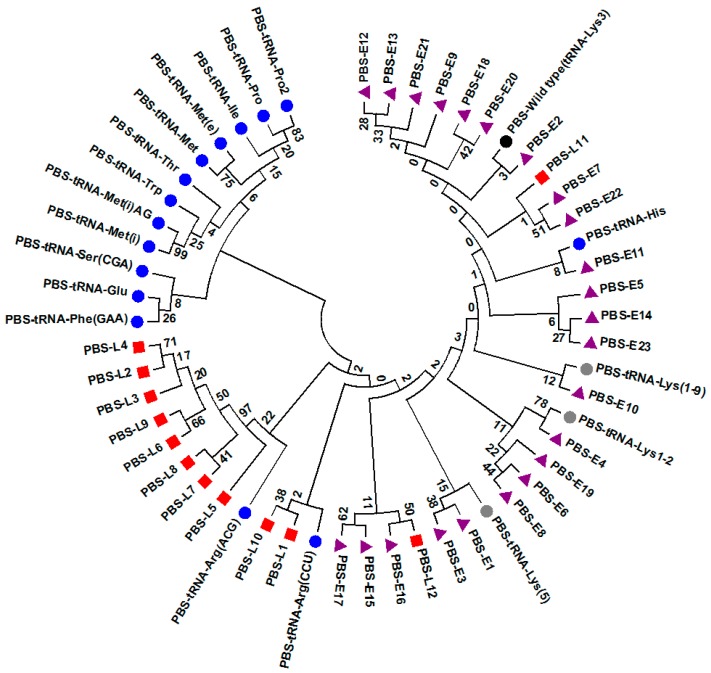
Molecular phylogenetic analysis of PBS sequence variants identified only in late (PBS-1 as PBS-L) or early (PBS-4 as PBS-E) seroconverters by maximum likelihood method with reference sequences.

Among the three sites studied, only PBS had sufficient supporting literature available to permit analysis for their potential functional significance. A phylogenetic tree was constructed containing PBS variant sequences only identified in late or early seroconverters, together with 19 PBS reference sequences that have been studied for their function (Figure 11). With the exception of tRNA^Lys,3^ and tRNA^Lys,5a^, none of the other tRNA molecules have been reported to be used as primers in naturally occurring HIV-1. Phylogenetic analysis showed that majority of the PBS sequence variants identified only in late seroconverters (PBS-1) co-clustered with PBS reference sequences utilizing tRNA^Arg^ molecules. Whereas, the PBS sequence variants identified only in early seroconverters (PBS-4) co-clustered with PBS wild type references PBS-tRNA^Lys,3^ and its variants PBS-tRNA^Lys1–9^, PBS-tRNA^Lys1,2^, PBS-tRNA^Lys(5)^, and PBS-tRNA^His^ (Figure 11).

The evolutionary history was inferred by using the maximum likelihood method based on the Tamura–Nei model [1]. The bootstrap consensus tree inferred from 1000 replicates [2] is taken to represent the evolutionary history of the taxa analyzed [2]. Branches corresponding to partitions reproduced in less than 50% bootstrap replicates are collapsed. Initial tree(s) for the heuristic search were obtained automatically by applying neighbor-join and BioNJ algorithms to a matrix of pairwise distances estimated using the maximum composite likelihood (MCL) approach, and then selecting the topology with superior log likelihood value. The analysis involved 54 nucleotide sequences. There was a total of 22 positions in the final dataset. Evolutionary analyses were conducted in MEGA6.

Note: reference sequences are marked with colored filled circles. The PBS sequences identified only from late seroconverters (PBS-1 as PBS-L) are marked with red filled square. The PBS sequences identified only from early seroconverters (PBS-4 as PBS-E) are marked with purple filled triangle.

## 4. Discussion

The outcome of exposure to HIV-1 is influenced by both host as well as pathogen derived genetic factors. HIV-1 late seroconversion has been observed in Pumwani sex worker cohort. Here, we investigate whether the late seroconversion is associated with specific subtypes and 5′LTR-leader sequence variants in this epidemiologically well-characterized cohort. We showed that the 5′LTR-leader sequence variants are dominated by clade A1 and D viruses in this cohort, and this is consistent with previous studies of Kenyan HIV infected patients [20,21,22]. We observed a significant difference in HIV-1 subtype distribution between late seroconverters and the early seroconverters. A significantly higher proportion of late seroconverters were infected by subtype A1 and D from Uganda. Two possibilities may explain this observation. One, viral subtypes from Uganda may differ in its ability to cause infection and exhibit superior replicative properties. Two, the late seroconverters may be infected while they were back in their home village during a break from sex work [17]. As none of the late seroconverters were from Uganda, it is possible that the migration of their clientele between Uganda and Kenya was responsible for the transmission of subtype A1.UG and D.UG. The predominance of the Uganda subtype in the late seroconverter population suggests a relationship between Ugandan viral origin and late seroconversion. HIV-1 subtypes originating in Uganda may be more infectious than their Kenyan counterparts, and comparative infectivity studies will need to be carried out to confirm this possibility. Moreover, the rates of disease progression of patients infected with Ugandan A and D subtypes could be examined and compared with that of patients infected with Kenyan subtypes A and D. In addition, other genetic factors unique to subtypes A1.UG and D.UG might play an important role in HIV-1 late seroconversion.

We also showed that unique sequence variants of PBS, SD, and PS exist in viruses infecting late seroconverters. Specific SD sequences were identified only in viruses from late seroconverters or early seroconverters. SD is essential to all splicing events in HIV-1 [36], and as such, the association of specific SD sequence variants with late seroconverters deserves specific attention. Functional studies, currently lacking, could address whether these specific SD sequence variants exhibit more efficient splicing activity. SD, PBS, and PS each have different roles in HIV-1 replication [28,29]. Our study showed that combinations of sequence variants from these sites associated significantly with late seroconverters or with the early seroconverters, suggesting a synergistic effect between these three functional sites. This appears also true in the combination of A1.KE or D with specific PBS and SD sequence variants infecting late or early seroconverters. Thus, both viral subtypes and PBS, SD, and PS sequence variants, play a role in late seroconversion. The interplay between the sequence variants of these sites and their effect on HIV-1 exposure outcome is not clear, and warrants further functional investigations.

Studies have shown that most of HIV viruses, including proviral sequences and virions in plasma samples, were defective. Our study is limited to the analysis of 5′LTR leader sequences; these diverse sequences may be associated with defective or non-defective HIV viruses. The identification of the specific PBS, SD, or PS variants, that exist only in LSC or EC, may provide a reasonable base to further investigate whether these specific sequence variants actually play a more important role in viral pathogenesis than the ones indicated by their population frequencies. In addition, studies have shown that defective viruses are known to drive HIV infection, persistence, and pathogenesis [58], and the data from our study provide another aspect of HIV pathogenesis.

Earlier studies done in our cohort suggested viral cytotoxic T lymphocyte escape variants were not likely to be the primary factors influencing HIV-1 late seroconversion, and pointed out potential links between loss or waning of HIV-1 epitope-specific responses after a break from sex work and late seroconversion [17]. The present study explored the phenomenon of late seroconversion further, and suggests that the process need not purely be immunological; virological factors, viz, PBS, SD1, PS variants and subtypes, could play important roles.

Analysis of potential functional implications of the PBS variant that were only identified in late or early seroconverters, based on the published data, showed that most of the PBS variants identified only in late seroconverters co-clustered with PBS sequence variants using tRNA^Arg^ as a primer for reverse transcription, whereas the PBS variants identified only in early seroconverters were co-clustered with the wild type PBS sequences using tRNA^Lys,3^, tRNA^Lys^ variants, or tRNA^His^ as a primer for reverse transcription. Studies have shown that HIV can replicate using either tRNA^His^ or tRNA^Lys1,2^ as primers [59,60,61,62,63,64], however, HIV mutants that use reverse transcription primers other than tRNA^Lys,3^ have reduced replication [65]. The only retrovirus that has been reported to use tRNA^Arg^ as a primer for reverse transcription is MuLV [66,67]. Analysis of the replication and stability of MuLVs with alternative PBSs revealed a preference for a PBS complementary to tRNA^Pro^, tRNA^Gly^, or tRNA^Arg^ [67]. The selection of tRNA^Arg^ for MuLV was probably facilitated, in part, by the multiple isoacceptors for tRNA^Arg^ [67]. Our study is the first to report that HIV PBS sequence variants identified only in late seroconverters, co-cluster with PBS sequences utilizing tRNA^Arg^ as a primer for reverse transcription. The PBS variants do not appear to belong to one specific subtype by interaction analysis (data not shown). Studies have shown that primer selection and viral translation, in particular, the synthesis of Gag-Pol, are linked [66,67]. How these specific HIV PBS variants, clustering with PBS sequences using tRNA^Arg^ as a primer for reverse transcription, contribute to the infection of women who were relatively resistant to HIV-1 infection, needs to be investigated.

The current study intends to investigate viral factors influencing HIV-1 late seroconversion observed in the Pumwani cohort. It is clear that the viral subtypes, as well as PBS, SD, and PS variants within the 5′ leader sequence, are associated with this clinical outcome, underscoring the importance of viral factors in the late seroconversion. Viral genotypes have been shown to exert profound influence over HIV-1 viral load [68]. Understanding why viruses of certain clades exhibit seemingly more infectiousness and pathogenicity will provide us with valuable information that could be used to help prevent HIV-1 infection. There is also a potential application for this knowledge to be used as clinical predictors that can serve to guide treatment decisions for patients. Successful inhibition of HIV-1 replication through small interfering RNA targeted to the PBS has been reported [69]. RNA transcripts containing HIV-1 PS sequences as HIV-1 antivirals have been explored [70]. To our knowledge, this is the first report of association of 5′LTR-leader sequence variation with HIV-1 late seroconversion, in addition to reporting the specific sequence variations in 5′ leader sequence region. The association of PBS, SD, and PS variants with LSC or EC identified in this study may help to find additional pharmaceutical targets, aiding the development of new anti-HIV therapeutics and HIV/AIDS prevention strategies.

## Figures and Tables

**Table 1:** Comparison of HIV-1 subtype distribution among late seroconverters and early seroconverters.

		HIV-1 Subtype	A1	A1.KE	A1.UG	B	C	D	D.UG	Total	Ave Seq./ind.	*p* Value
A	All 142 individuals	No.	30	2162	135	97	263	945	46	3678		
		%	0.8	58.8	3.7	2.6	7.2	25.7	1.3	100		
B	Late seroconverter (*n* = 20)	No.	0	452	91	0	0	211	46	800	40 (19–47)	<0.0001
		%	0	56.5	11.4	0	0	26.4	5.8	100		
	Early seroconverter (*n* = 122)	No.	30	1710	44	97	263	734	0	2878	30 (11–75)	
		%	1.0	59.4	1.5	3.4	9.1	25.5	0	100		

**Table 2:** PBS, SD1, and PS sequences listed in Figure 3, Figure 4 and Figure 5.

Group	Sequences	Subtypes	Frequency in EC or LSC
PBS-1	GAGAAAGTTAACAGGGAC, GCGAAAGATAACAGGGAC, GCGAAAGCTAACAGGGAC, GCGAAAGTTAATAGGGA-C, GCGAAAGTTAACAGGGAC, GCGAAAGTTAATAGGGAC, GCGAGAGTTAACAGGGAC, GTGAAAGTTAACAGGGAC, TGGCGCCCGAAGAGGGAC, TGGCGCCCAAACAGGGAC, TGGGCGCCCCAACGGGGAC, TGG-GCCCGAACAGGGAC	A1.KE (1.1%), A1.UG (93.67%), D (3.2%), D.UG (2.1%)	0/122 EC, 5/12 LSC
PBS-4	TG-GCGCC-GAACAGGGA, TGGCGCCCGAACAGGGTAC, TGGCGCCGGAACAGGGAC, TGGCGCCCGACGTGGGGC, TGGCGCCCGAACCGGGAC, TGGCGCCCGACCAGGGAC, TGGC-CCCGAACAGGAAC, TGGCGCCCGA-CAGGGAC, TG-CGCC-CGAACAGGGAC, TGGCGCCCGTACAGGGAC, TGGCGCCCGAACAGGGTTC, TGGCGCC-GAACAGGGA, TGGCGACCGAACAGGGAC, TGGCGCCCGAAT-GGGAC, TGGCGCCCCAACAGGGAC, TGGCGCCC-AACAGGGAC, TGGCGCCCCGAACAGGGAC, -GCGCCC-GAACAGGGAC, TGGCGCCCGATCAGGGAC, CGGCGCCCGAACAGGGAC, TGGC-GCC-GAACAGGGAC, TGGCGCCCGAACAGG-AC, TG--GCGCCCGAACTGGGA	A1 (2.2%), A1.KE (38%), B (2.2%), C (12.0%), D (42%)	36/122 EC, 0/20 LSC
SD1-1	AAGGCGAGTAC, GAGGTGAGTAC, CTAGGTGAGTAC, CTAGGTGGGTAC, CTGGTAGGTGC, ACGGTGTTTAC, ATGGTGAGTAC, ACGGTGTGTAC, ACGGTGAATAC	A1.KE (92.4), D (6.2%), D.UG (1.4%)	0/122 EC, 6/20 LSC
SD1-5	ACGGTAAGTAC, CGGGGGAGTAC, TTGGTGAGTAC, CTGG-TGAGTGC, CCGGTGAGCAC, CTGGGTGAGTAC, CAGGTGAGTGC, CAGGTGAGTAC, CTGGGGAGTAC, GTGGGTGAGTAC, CTGGTGAATAC, CTGGTGAGTGT, CGGGTGAGTAC, ACGGTGAGTGC, CTGGTGAGTGC	A1 (1.4%), A1.KE (90%), B (1.4%), C (2.9%), D (4.3%)	15/122 EC, 0/20 LSC
PS-1	AGTG, GGAC, CGAG, GGCG, AGGG	A1.KE (50%), A1.UG (16.7%), D (33.3%)	0/122 EC, 6/20 LSC
PS-3	GGAA, GAAG, GGAT, AGAG, ?	A1.KE (66.8%), A1.UG (1.3%), C (24.1%), D (7.8%)	30/122, EC, 4/20 LSC

Notes: ? denotes lack of sequence.

**Table 3:** Specific subtype, PBS, and SD variant combinations in late or early seroconverters.

Subtypes or PBS	SD or PBS	Seroconverter
TGGCGCCCGAACAGGGGC TGGCGCCCGAACAGGGTC TGGCGCCCGAATAGGGAC TGGCGCCCGAACAGGAAC TGGCGCCCGCACAGGGAC? (PBS-2)	CTGGTGAGTAC AAGGTGAGTAC ACGGTGTTTAC ACAGTGAGTAC	LSC
	CAGGTGAGTAC CAGGTGAGTGC	EC
TGGCGCCCGAACAGGGAC TGGCGCCCGAACAGAGAC TGGCGCC-GAACAGGGAC TGGCGCCCGAACGGGGAC TGGC-CCCGAACAGGGAC TGGCCGCCCGAACAGGGAC TG-CGCCCGAACAGGGAC TGGCGCCCGAACTGGGAC (PBS-3)	AAGGCGAGTAC ACGGTGAATAC ATGGTGAGTAC CTAGGTAGGTGC CTAGGTGGGTAC CTGGTAGGTGC GAGGTGAGTAC	LSC
	ACGGTGAGTGC ACGGTAAGTAC CAGGTGAGTAC CAGGTGAGTGC CCGGTGAGCAC CGGGGGAGTAC CGGGTGAGTAC CTGGTGAGTGT CTGGGGAGTAC CTGGGTGAGTAC CTGG-TGAGTGC CTGGTGAATAC GTGGGTGAGTAC TTGGTGAGTAC	EC
Subtype D	AAGGCGAGTAC ACGGTGAATAC ACGGTGTGTAC ACGGTGTTTAC ATGGTGAGTAC ?	LSC
	ACGGTAAGTAC ACGGTGAGTGC CCGGTGAGTAC CTAGTGAGTAC CTGGTAAGTAC CTGGTGAATAC CTGGTGAGCAC CTGGCGAGTAC	EC
Subtype D	TGGCGCCCGAACAGGGTC TGGCGCCCCAACGGGGAC TGGCGCCCGAACAGGAAC TGG-GCCCGAACAGGGAC TGGCGCCCAAACAGGGAC TG-CGCCCGAACAGGGAC TGGCCGCCCGAACAGGGAC (D-PBS-1)	LSC
	TGGCGCCGGAACAGGGAC TGGCGCCCGAACAGGGTAC TGGCGCCCGACGTGGGGC TGGCGACCGAACAGGGAC TGGCGCCCGAACCGGGAC TGGCGCCCGTACAGGGAC TGGC-CCCGAACAGGGAC TGGCCGCCCGATCAGGGAC TG-CGCC-CGAACAGGGAC TGGCGCCCCGAACAGGGAC (D-PBS-3)	EC
A1.KE	CTAGGTGAGTAC CTAGGTGGGTAC CTAGTGAGTAC CTGGTAGGTGC (A1.KE-SD-1)	LSC
	ACGGTGAGTAC ACGGTGAGTGC CAGGTGAGTAC CAGGTGAGTGC CCGGTGAGTAC CGGGGGAGTAC CTGGGTGAGTAC CTGGTGAGTGT CTGG-TGAGTGC TTGGTGAGTAC ? (A1.KE-SD-4)	EC

Note: ‘?’ denotes lack of sequence.

**Table 4:** Later seroconverters with HIV subtypes, PBS, SD, PS variants that are enriched or only identified in LSC.

mlno	PBS1	SD1	PS1	PBS-2/SD-1	PBS2/A1.UG	PBS-3/SD-1	PBS-3/SD-2	SD2	A1-UG/D.UG	A1.KE/SD1	D-SD-1	D-PBS-1
37			+									
58	+	+				+		+	+			
290				+	+				+			
452				+			+	+				
546		+				+				+		
768	+		+	+					+			
814		+				+				+		
825												
888	+	+	+	+		+					+	+
890	+		+	+			+	+				
1072				+	+				+			
1102		+	+	+		+						
1232							+	+				
1248												
1250	+			+				+				+
1287		+		+		+		+			+	
1430				+								
1626			+				+	+				
1707												
1730												

Note: ‘+’ denotes presence of indicated sequences or subtypes, or their combinations.

**Table 5:** HIV-1 subtype classification of 5′LTR-leader variants of viruses infecting late seroconverters.

mlno	A1.KE	A1.UG	D	D.UG
37	*			
58				*
290		*		
452	*			
546	*			
768	*	*		
814	*			
825	*		*	
888			*	
890	*			
1072	*	*		
1102			*	
1232	*			
1248			*	
1250			*	
1287			*	
1430	*		*	
1626	*			
1707	*			
1730	*			

Note: ‘*’ denotes presence of indicated subtypes.

## Supplementary Materials

The following are available online at www.mdpi.com/1999-4915/10/1/4/s1.
