# Supplementary material for: HIV-1 Subtypes and 5′LTR-Leader Sequence Variants Correlate with Seroconversion Status in Pumwani Sex Worker Cohort
Source: Viruses. 2017 Dec 23;10(1):4. doi: 10.3390/v10010004 (PMC5795417; doi:10.3390/v10010004)

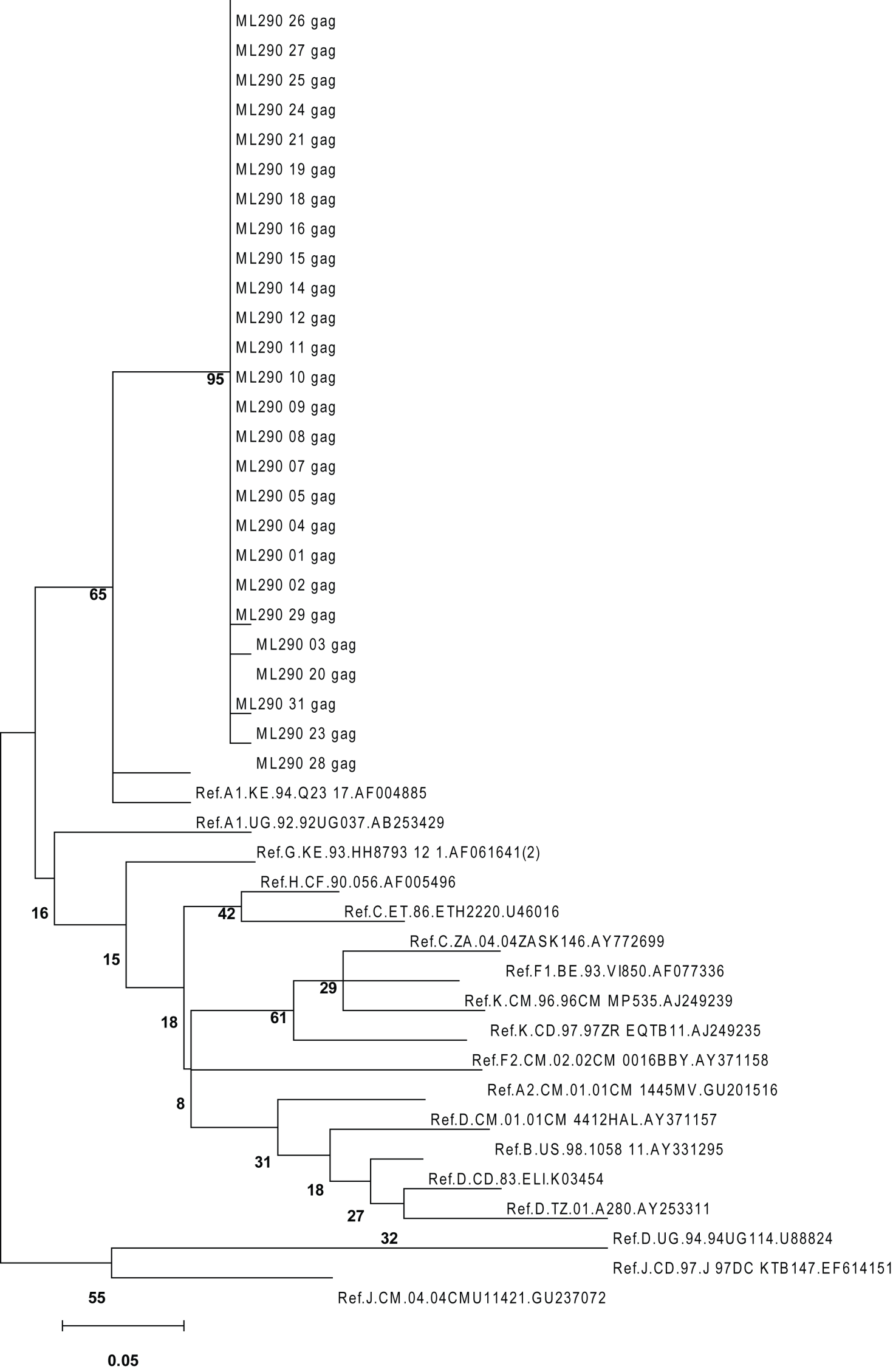


**Supplemental Figure 1**. Phylogenetic analysis of p17 sequences of HIV viral sequence clones of patient ML290. The p17 sequences showed the consistent HIV subtype assignment as the partial 5’LTR sequences.

**Supplemental Figure 2**. Phylogenetic analysis of p17 sequences of HIV viral sequence clones of patient ML825. The p17 sequences showed the consistent HIV subtype assignment as the partial 5’LTR sequences.


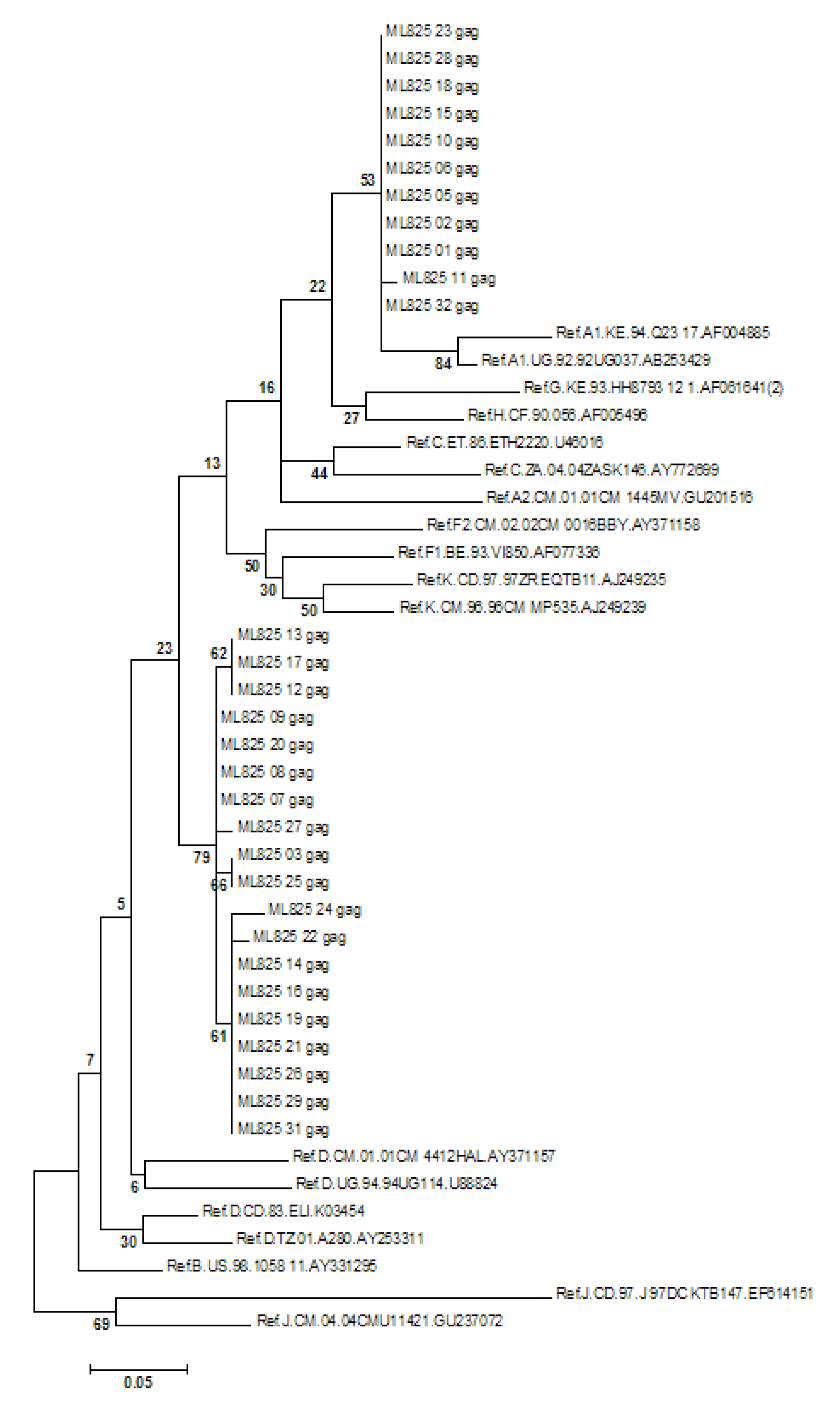

Supplement: Supplementary file 1 [file viruses-10-00004-s001.docx]
